# Deterministic Development of Soil Microbial Communities in Disturbed Soils Depends on Microbial Biomass of the Bioinoculum

**DOI:** 10.1007/s00248-023-02285-9

**Published:** 2023-08-25

**Authors:** Yuri Pinheiro Alves de Souza, Michael Schloter, Wolfgang Weisser, Stefanie Schulz

**Affiliations:** 1 Helmholtz Zentrum München, Research Unit Comparative Microbiome Analysis, Neuherberg, Germany; 2 Technische Universität München, TUM School of Life Science, Chair of Environmental Microbiology, Freising, Germany; 3 Technische Universität München, TUM School of Life Science, Chair of Terrestrial Ecology, Freising, Germany

**Keywords:** Soil microbiome, Metabarcoding, Soil disturbance, Recolonization, βNTI

## Abstract

Despite its enormous importance for ecosystem services, factors driving microbial recolonization of soils after disturbance are still poorly understood. Here, we compared the microbial recolonization patterns of a disturbed, autoclaved soil using different amounts of the original non-disturbed soil as inoculum. By using this approach, we manipulated microbial biomass, but did not change microbial diversity of the inoculum. We followed the development of a new soil microbiome after reinoculation over a period of 4 weeks using a molecular barcoding approach as well as qPCR. Focus was given on the assessment of bacteria and archaea. We could show that 1 week after inoculation in all inoculated treatments bacterial biomass exceeded the values from the original soil as a consequence of high dissolved organic carbon (DOC) concentrations in the disturbed soil resulting from the disturbance. This high biomass was persistent over the complete experimental period. In line with the high DOC concentrations, in the first 2 weeks of incubation, copiotrophic bacteria dominated the community, which derived from the inoculum used. Only in the disturbed control soils which did not receive a microbial inoculum, recolonization pattern differed. In contrast, archaeal biomass did not recover over the experimental period and recolonization was strongly triggered by amount of inoculated original soil added. Interestingly, the variability between replicates of the same inoculation density decreased with increasing biomass in the inoculum, indicating a deterministic development of soil microbiomes if higher numbers of cells are used for reinoculation.

**Supplementary Information:**

The online version contains supplementary material available at 10.1007/s00248-023-02285-9.

## Introduction

Soil is one of the most diverse environments on the planet [1]. Its heterogeneity leads to complex three-dimensional spaces where microbes interact and compete for nutrients and niche formation, promoting species diversification and interaction [2]. The microbial colonization and community assembly of soils is driven by four main processes, which are diversification, dispersal, selection, and drift [3]. Among those, diversification and dispersal are related to the introduction of new species, while drift and selection determine the relative abundance and turnover of species [4]. The latter is strongly related to the nutrient status of the soil and thus the growth rate of microbes.

Species selection is governed by both deterministic and stochastic factors and interactions [5]. The deterministic share of this process, also called “niche-based” mechanisms includes environmental filtering, which is the interaction between individuals, the surounding abiotic environment and interspecific interactions and trade-offs, while the stochastic aspect, is governed by “neutral processes”, including unpredictable disturbances, life and death events, random dispersal and colonization, extinctions, and ecological drift [5, 6]. Regarding dispersal, Nemergut et al. (2013) clarified that dispersal and migration must be clearly distinguished, since the chance to reach a site (dispersal) does not guarantee the successful establishment on the site (migration). Thus, rather environmental filters and niche competition, which are related to the process of selection [7], might determine the successful establishment of microbes.

In this context, it has been demonstrated previously that successful invasion of non-native species relies on the number of available niches [8, 9] and is negatively correlated with diversity, which is known as the diversity–invasiveness relationship [3]. Chiba et al. (2021) demonstrated that litter born microbes were only able to invade soil, if microbial diversity of the soil community was low, as achieved by autoclaving. Additionally, the colonization sequence of different microbial taxa seems to be a critical factor for further community development [9]. Causes for such divergence include facilitation or inhibition through microbial interaction [3], or changes of environmental conditions [10] like depletion of easily available nutrients. Delmont et al. (2014) [11] demonstrated that environmental filtering determines microbial colonization more than the composition of the inoculum community. They performed a cross-incubation experiment where they inoculated sterilized soil with its own initial microbial community or that from another soil origin. In general, in the case of sterilized soils, an endogenous heterotrophic succession can be expected [12] where nutrients released from dead cells will feed copiotrophic bacteria in the early development stages.

The knowledge about microbial community assembly and colonization is used in different field applications as well as laboratory setups including reclamation strategies, where microbial communities of natural soils from the same area are used as bioinoculum to direct the soil reclamation towards the previous natural situation [13]. Recently, it has been also suggested to inoculate microbiota from highly diverse soil ecosystems into soils, which are degraded as a result of intensive agricultural practice, to re-establish ecosystem services at such sites [14]. In several laboratory experiments often sterile soils have been used as substrate, which have been re-inoculated with differently diverse communities [15, 16]. Often differences in microbial diversity are achieved by using a distinction by dilution technique [8, 12], which is in most cases confounded by differences in microbial biomass of the inoculum. In addition, the ratio between soil and inoculum might determine the dynamics of community assembly and the likelihood of successful invasion of alien species. The question of what drives community development in disturbed soils between the diversity of inoculated microbiota and the inoculum microbial biomass is still not fully answered.

To test the consequences of inoculum amount (microbial biomass of the inoculum) for the recovery of microbial communities, we performed an incubation experiment with disturbed soil, which was achieved by successive autoclaving. The disturbed soil was either inoculated with 10%, 2%, or 1% of the original soil community (treatments) or left without an inoculum (non-inoculated control). Thus, the introduced microbial diversity was comparable between treatments and only the microbial biomass differed. These treatments were incubated 5 weeks under constant water content (50% maximal water holding capacity) and temperature (20 °C). The development of bacterial and archaeal biomass, diversity and community assembly were compared with the original soil (original soil control), which was incubated alongside. We combined quantitative PCR measurements, diversity analysis using metabarcoding approaches and community assembly analysis based on the calculation of nearest taxon index [17], which is a measure of phylogenetic relatedness using the 16S rRNA gene as a marker.

We hypothesized that (1) differences in microbial biomass of the inoculum will significantly impact microbial colonization pattern of disturbed soils. (2) Increasing biomass will induce more deterministic development of the soil microbiome whereas lower diversity and cell numbers in the inoculum will result in stochastic development of the soil microbiome. (3) The new established microbial community will be dominated by copiotroph bacteria, possibly spore forming bacteria, making use of necromass nutrients present in soil and differ from the community composition of the original soil.

## Material and Methods

### Soil Sampling and Sterilization

Soil samples were taken in June 2020 from “The Jena Experiment” field side (Roscher et al. 2004; Weisser et al. 2017 - http://the-jena-experiment.de/), which is located in Jena (Thuringia, Germany, 50°55′N, 11°35′E, 130 m a.s.l.) on the floodplain of the Saale river (altitude 130 m a.s.l.). The mean annual air temperature is 9.9 °C (1980–2010), and mean annual precipitation is 610 mm [18]. The Jena Experiment is composed of 82 plots with dimensions of 20 × 20 m where plant diversity has been manipulated since more than 20 years. The soil for our experiment was taken outside the treatment plots, reflecting the original soil where the experiment has been built on. The soil is classified as an Eutric Fluvisol (World Reference Base for Soil Resources 2015 [19]) with a pH value range from 7.1 to 8.4 and C_org_ 5–33 g C kg^−1^ [20]. A total of 50 kg fresh soil was sampled from the top 20 cm of a 1 m^2^ area, using a shovel, and transported to the lab. For homogenization, the soil was sieved to 5 mm. The soil was split into two parts: one part to be disturbed and the other part to be used as control for the natural soil as well as to generate the inoculum. Soils were kept at 4 °C until further processing.

Disturbed soil was obtained by autoclaving. Autoclaving was done at 130 °C and 1.5 ATM for 1 h. After autoclaving the soil was incubated at 4 °C for 1 week to allow for potential spore germination and tested for successful sterilization. Therefore, 0.5 g soil and 100 μL of 0.8% NaCl sterile saline buffer were mixed. The obtained soil slurry was diluted 1:10, 1:100, and 1:1000, and 100μL of each dilution was plated on R2A [18] agar plates. Plates were incubated for a week at room temperature, and growth was evaluated at the end of this period. This cycle of autoclaving, incubation and testing for sterility was repeated four times until no microbial growth on R2A medium was observed (Fig. S1).” After four rounds of autoclaving, no microbial growth on R2A medium was observed anymore.

### Experimental Design

To evaluate the soil microbial recolonization after autoclaving, disturbed soil was reinoculated with original soil in three different proportions: 1:10 (10% inoculation), 1:50 (2%), and 1:100 (1%) by mixing. The amount of inoculum was calculated based on soil dry weight (w/w). In addition, both non-autoclaved soil (termed *Original*) and the autoclaved non-inoculated soil (termed *No inoculum*) served as controls alongside the experiment. Per treatment 15 g of the soil mixtures were incubated in open, 50 ml Falcon Tubes (Universal Medical-Germany) at 20 °C in the dark for 4 weeks. During the incubation, soil moisture was kept constant at 50% of maximum water holding capacity (mWHC) by watering every second day. Samples were taken at the beginning (T0) and after one (T1), two (T2), and 4 weeks (T4) of incubation from three independent replicates per treatment. Samples were immediately frozen at −20 °C for DNA analysis. Overall, 60 samples (5 treatments × 3 replicates × 4 samplings) were analyzed.

### Dissolved Organic Carbon

Dissolved organic carbon (DOC) was extracted by incubating 4 g of soil in 20 ml of 0.01 M CaCl_2_ solution for 40 min on an overhead shaker [21]. Afterwards, samples were filtered using cellulose filter papers (595 ½ filter papers, Whatman–Germany). The DOC concentration of the extracts was measured with a DIMA-TOC 100 (Dima Tec, Germany).

### DNA Extraction

Soil DNA was extracted from 0.5 g of fresh soil following a Phenol/Chloroform/Isoamyl alcohol method (modified from Pommerenke and Friedrich, 2007). The sample lysis was done using Lysing Matrix E tubes (MP Biomedicals™, Germany). The bead beating was done using the TissueLyser II bead beater (QIAGEN®, Germany) at a frequency of 15 Hz during 2 min. Resulting DNA was quantified by Qubit fluorometric system (Thermo Fisher Scientific, Germany) using the broad range assay kit. The DNA quality was checked using the Nanodrop photometric system (Thermo Fisher Scientific, Germany) and by agarose gel electrophoresis. The extracted DNA was stored at −20 C° until usage. To exclude contaminations during DNA extraction a blank control without soil was included.

In addition to the normal DNA extraction, a smaller test was performed to estimate how much DNA in the original soil derived from intact cells. We used propidium monoazide (PMA), a photo-reactive DNA-binding dye which, when exposed to light, degrades extracellular DNA [22]. Therefore, a set of parallel DNA extractions was performed. Those comprise the original soil (−PMA), original soil treated with PMA (+PMA), a “dead” soil control (dead−PMA) and a “dead” soil treated with PMA (dead+PMA). The “dead” soil control was included to determine the capability of PMA to remove DNA from the samples, being used to calculate the efficiency of extracellular DNA removal. The “dead” soil samples were heated at 90 °C for 1 h prior to DNA extraction, in order to kill vegetative cells on the samples. The test was conducted in triplicate for each treatment. Soil samples with PMA (0.5 g) were incubated in the dark with 25 μM of PMA (Biotium-Germany) for 10 min, and then exposed to white light for 25 min. DNA was extracted as described above. The percentage of DNA coming from dead and/or damaged cells was calculated by follows:

$$\textrm{ExtracelularDNA}\%=\left[\left(1-\left(\frac{\textrm{dead}+\textrm{PMA}}{\textrm{dead}-\textrm{PMA}}\right)\right)\times \left(1-\left(\frac{+\textrm{PMA}}{-\textrm{PMA}}\right)\right)\right]\times 100$$

### Quantitative PCR of Bacteria and Archaea

Bacterial and archaeal abundance was determined by a SybrGreen based absolute quantification using a 7300 Real-Time PCR System (Applied Biosystems, Germany). To quantify bacteria, the primer pair FP 16S and RP 16S [23] was used; for archaea the primer pair rSAf(i) [24] and 958r (Bano et al. 2004). Each PCR reactions contained 12.5 μL Power SYBR™ Green PCR Master Mix (Thermo Fisher Scientific, Germany), 1 μL of each primer with 10 pmol μL^−1^, 0.5 μL of 3% BSA, 2 μL of extracted DNA, and 8.0 μL of DEPC treated water. Thermal profiles are summarized in Table 1. As standard curve serial dilutions (10^3^ to 10^9^ copies per μL^−1^) of plasmids containing the 16S rRNA gene fragment of *Pseudomonas putida* for bacteria and *Methanobacterium* sp. for archaea were used in three technical replications. Besides three replicates per standard, three no template controls were included in each run. The obtained copy number was subtracted from samples and controls.

**Table 1:** Thermal profiles for qPCR of the 16S rRNA gene of bacteria and archaea

Target Gene	Thermal profile	No cycles
16S rRNA bacteria	95 °C—10 min	1
	95 °C—45 s/58 °C; 45 s/ 72 °C—45 s	35
16S rRNA archaea	95 °C—10 min	1
	95 °C—20 s/55 °C; 60 s/72 °C—60 s	5 (touchdown: −1 °C per cycle)
	95 °C—20 s/58 °C; 60 s/72 °C—60 s	40

To exclude any inhibitory effects of co-extracted substances, a dilution test was performed, which identified a 1:32 dilution as sufficient. The specificity of the PCR product was ensured by doing a melting curve analysis as well as an agarose gel at 1.5%, running for 40 min at 120 V and 400 mA. Final copy numbers were calculated by normalizing the number of copies per μL to g of dry soil. The respective qPCR efficiency (calculated with the formula Eff = [10^(1/slope)^−1]) and *R*^2^ for bacteria and archaea ranged between *R*^2^ 0.997 and 0.982 and efficiency 76.47% and 71.12% for bacteria and; *R*^2^ 0.998 and 0.997 and efficiency 84% and 85.4% for archaea.

### Amplicon Sequencing and Bioinformatics

To analyze bacterial and archaeal diversity, a metabarcoding approach was used following the “16S Metagenomic Sequencing Library Preparation” protocol (Illumina, San Diego, CA, USA) and quality guidelines recommended by Schöler et al. (2017). Bacteria and archaea were targeted together by using the universal primer pair 515FB and 806RB [25, 26] with Illumina adapters. Each reaction contained 12.5 μL NEB Next High-Fidelity Master Mix (Thermo, Germany), 0.5 μL of each primer at 10 pmol μL^−1^, 2.5 μL of 3% BSA, 1 μL of 5 ng μL^−1^ diluted DNA, and 8 μL of DEPC treated water. The thermal profile was the following: 98°C for 1 min, followed by 23 cycles of 98 °C for 10 s, 55 °C for 30 s, and 72 °C for 30 s, ended by final extension of 72 °C for 5 min. Afterwards, samples were purified with the MagSi-NGSprep Plus Beads (ratio 0.8 beads: 1 sample); according to the manufacturers protocol and quantity of the PCR, product was measured using the Quant-iT PicoGreen dsDNA Assay Kit (Thermo Fisher Scientific, Germany). Purified samples were indexed using Nextera® XT Index Kit v2 (Illumina, San Diego, CA, USA) and again purified with MagSi-NGSprep Plus Beads (ratio 0.8 beads: 1 sample). Quality assessment and final quantification of the indexed fragments was done via Fragment Analyser (Agilent, Germany). High-quality DNA was diluted to 4 nM and sequenced on Illumina MiSeq using MiSeq Reagent v3 (600 Cycle) kit. 5 pM, 20% PhiX was loaded alongside the samples.

Raw sequences were demultiplexed based on the associated barcodes and adapters were removed using AdapterRemoval version 2.3.1 [27]. Afterwards datasets were processed using Qiime2 version 2021.2 and the plugin DADA 2 version 1.18 [28]. The sample processing includes denoising using denoise-paired option with the following parameters: --p-trim-left-f 20; --p-trim-left-r 20; --p-trunc-len-f 240; --p-trunc-len-r 200; --p-max-ee-f 4; --p-max-ee-r 4. Taxonomic assignments were done against SILVA database (version 138) using *classify-consensus-blast* option in default parameters. The raw sequencing reads were uploaded to NCBI sequencing read archive under the bioproject number PRJNA937438.

### Statistical Analysis

The table of amplified sequence variants (ASVs) was exported to R, where Phyloseq version 1.42.0 [29] and Vegan version 2.6-2 packages were used to construct rarefaction curves to observe sequencing coverage, to normalize the number of reads in each sample using median sequencing depth, plot relative abundance and ordination plots (NMDS with Bray-Curtis dissimilarity distance). Furthermore, the packages were used to calculate alpha diversity metrics (Observed ASVS, Shannon diversity and Evenness). Those metrics were used as response variable for experimental design and statistical significances across the treatment was calculated via ANOVA. Pairwise comparison was done using Tukey post hoc test for multiple comparisons of means. Additionally, to get values for bacteria and archaea separately, bacterial and archaeal reads were filtered manually from a phyloseq object and analyzed the same way. From a total of 7820 ASVs, distributed in 3.224.377 reads, 7764 ASVs (3.185.379 reads) were assigned as bacteria and 56 ASVs (38.998 reads) were from archaea. We also used PERMANOVA to calculate the effect size and significance over beta diversity, using as input a Bray-Curtis dissimilarity matrix. Prior to statistical testing, data was checked for normality via the Shapiro-Wilk tests and Q-Q plots.

The Qiime2 plugin SourceTracker version 1.0.1 [30] was used with default parameters to track the origin of ASVs in different treatments. The original soil at T0 was treated here as source. The sink was the re-inoculated soils.

The normalized dataset was used to plot the taxonomical composition of the most abundant taxa in a form of a heatmap using the pheatmap package (version 1.0.12). ASVs with abundance of 5% or greater in at least one of the samples were filtered using the *filter taxa* function on phyloseq package. Dendrograms were drawn using Ward’s Hierarchical Clustering.

To assess the phylogenetic relationship, we calculated the mean-nearest-taxon-distance (MNTD) and the nearest-taxon-index (NTI) [17] using “mntd” and “ses.mntd” implemented in the package “picante” version 1.8.2 [31]. This procedure allows to determine whether a given community (input is a phylogenetic tree generated by Qiime2) is more phylogenetic related than expected by chance, when compared to a random version of this same community (*null model*) [17]. We use this metric to estimate whether microbial communities’ assembly stochastically (NTI less than −2) or deterministically (NTI greater than +2). To generate the underlying phylogenetic tree, Qiime2 was used by applying the *alignment mafft* function for alignment, *alignment mask* to mask ambiguously aligned regions, *fasttree* for tree generation, and *phylogeny midpoint-root* for tree rooting.

## Results

### Recovery of Bacterial and Archaeal Biomass After Reinoculation of Disturbed Soil

Bacterial abundance was significantly influenced by time (bacteria: *p* = 0.003, *F* = 5.247) while archaeal abundance not (*p* = 0.184, *F* = 1.673). The abundances of both groups were significantly impacted by the dilution treatment (bacteria: *p* = 0.00745, *F* = 3.918; archaea: *p* = 1.79e-15, *F* = 40.927). The Tukey post hoc test (Table S1) indicates that bacterial abundance significantly differed between T0 and T1 (*p =* 0.009) and T0 and T4 (*p* = 0.019). Across all treatments, significant differences of bacterial abundance were found when samples with no-inoculum and inoculation level 1:10 (*p* = 0.016) were compared, while for archaea the differences were between no-inoculum samples and inoculation level 1:10 (*p* = 0.0092348) as well as between original soil and all inoculation levels, being 1:10 (*p* < 0.00001), 1:50 (*p* < 0.00001), 1:100 (*p* < 0.00001), and non-inoculated soils (*p* < 0.00001).

**Fig. 1:**
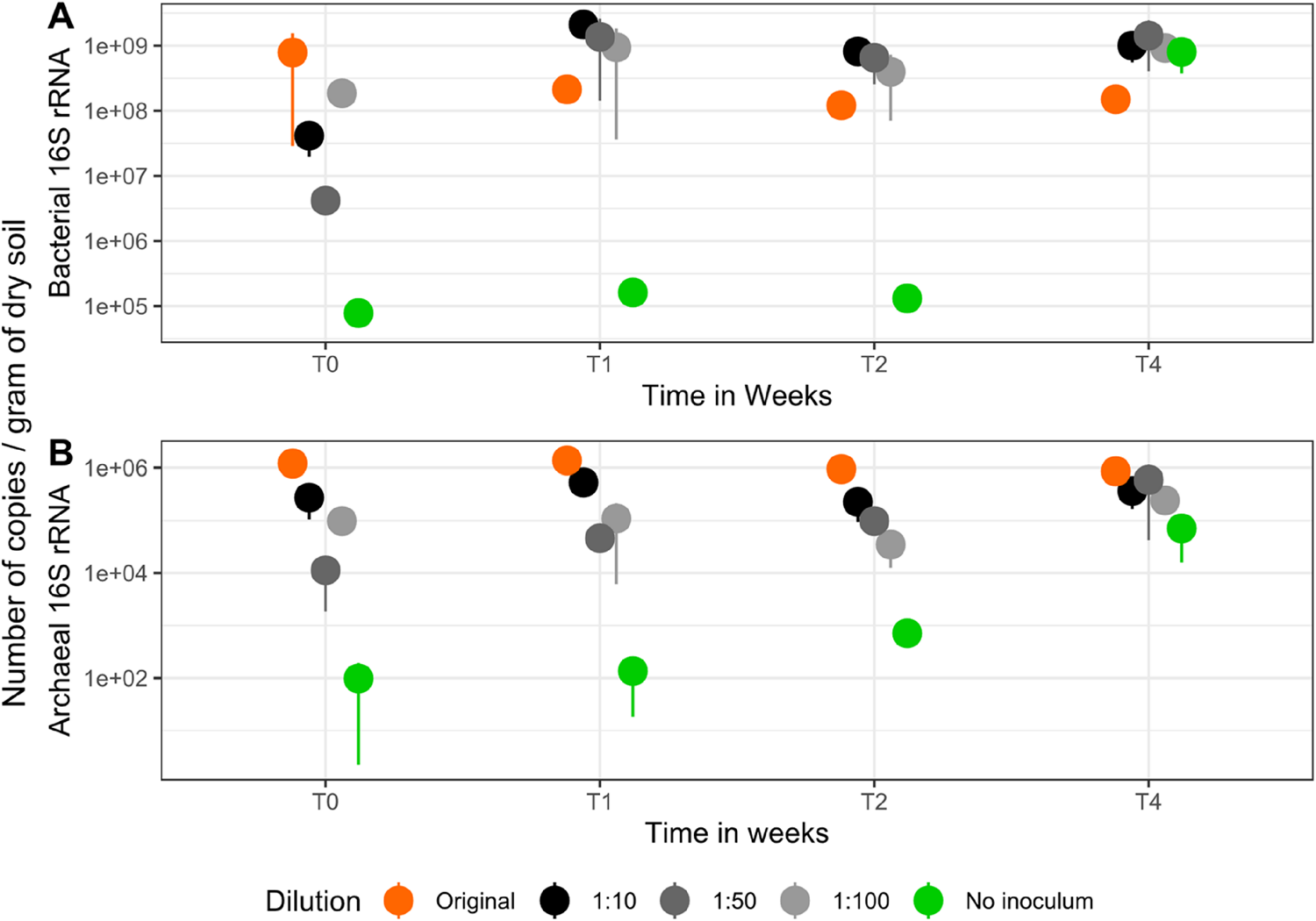
Mean values of the absolute quantification of 16S rRNA gene copies per g^−*1*^ dry soil for bacteria (**A**) and archaea (**B**). Treatments are color coded as follows: original soil (orange), the reinoculated dilutions 1:10 dilution (black), 1:50 dilution (dark grey), 1:100 dilution (light grey), and the soil without inoculum (green) soil across the experimental period (T0–T4). Error bars are standard deviation (*n* = 3). *Y*-axis is presented in logarithmic scale

Bacterial and archaeal abundance in original soils remained stable over the entire period of the experiment ranging between 1.2* 10^8^ to 9* 10^8^ copies g^−1^ of dry soil for bacteria and 9* 10^5^ to 2.3* 10^6^ copies g^−1^ of dry soil for archaea (Fig. 1A and B). Already after 1 week of incubation (T1), bacterial abundance of the inoculated treatments was significantly higher than that of the original soil and remained stable until the end of the experiment, at average 1.8* 10^9^ copies g^−1^ of dry soil, independent from inoculation level. In contrast, in the non-inoculated disturbed soil treatment, bacterial abundance was lowest at T0 (7.8* 10^4^ copies g^−1^ of dry soil), indicating that some microorganisms or their DNA remained in the soil after 4 steps of autoclaving. However, recovery of microbial biomass in non-inoculated soils was much slower compared to the inoculated treatments and reached comparable values only after 3 weeks of incubation (T3).

Abundance of archaea was at least 2 orders of magnitude lower than the bacterial counterpart over the complete duration of the experiment in all treatments. In contrast to bacteria, we observed that the amount of inoculated soil positively affected the community recovery since the 1:10 dilutions lead to a consistently higher number of copies (average 5.5e+05 copies g^−1^ of dry soil) compared to the treatment level 1:50 (average 2.0e+05 copies g^−1^ of dry soil) and 1:100 (average 1.5 e+05 copies g^−1^ of dry soil). Values in the original soil were always higher independent from the biomass of the inoculum (average 1.4 e+06 copies g^−1^ of dry soil) (Fig. 1B).

The viability PCR (PMA PCR) indicated that 23.9% of DNA present in the original soil samples was derived from non-intact cells (see Fig. S5).

### Recovery of Prokaryotic Alpha Diversity After Reinoculation of Disturbed Soil

The 16S rRNA amplicon sequencing yielded a total of 3.325.064 high quality reads. After filtering, denoising, merging, and chimeral removal 3.267.439 reads remained, which were normalized to 54.515 reads per sample. Rarefaction analysis revealed that the number of reads was enough to cover bacterial and archaeal diversity in the samples (Fig. S3).

**Fig. 2:**
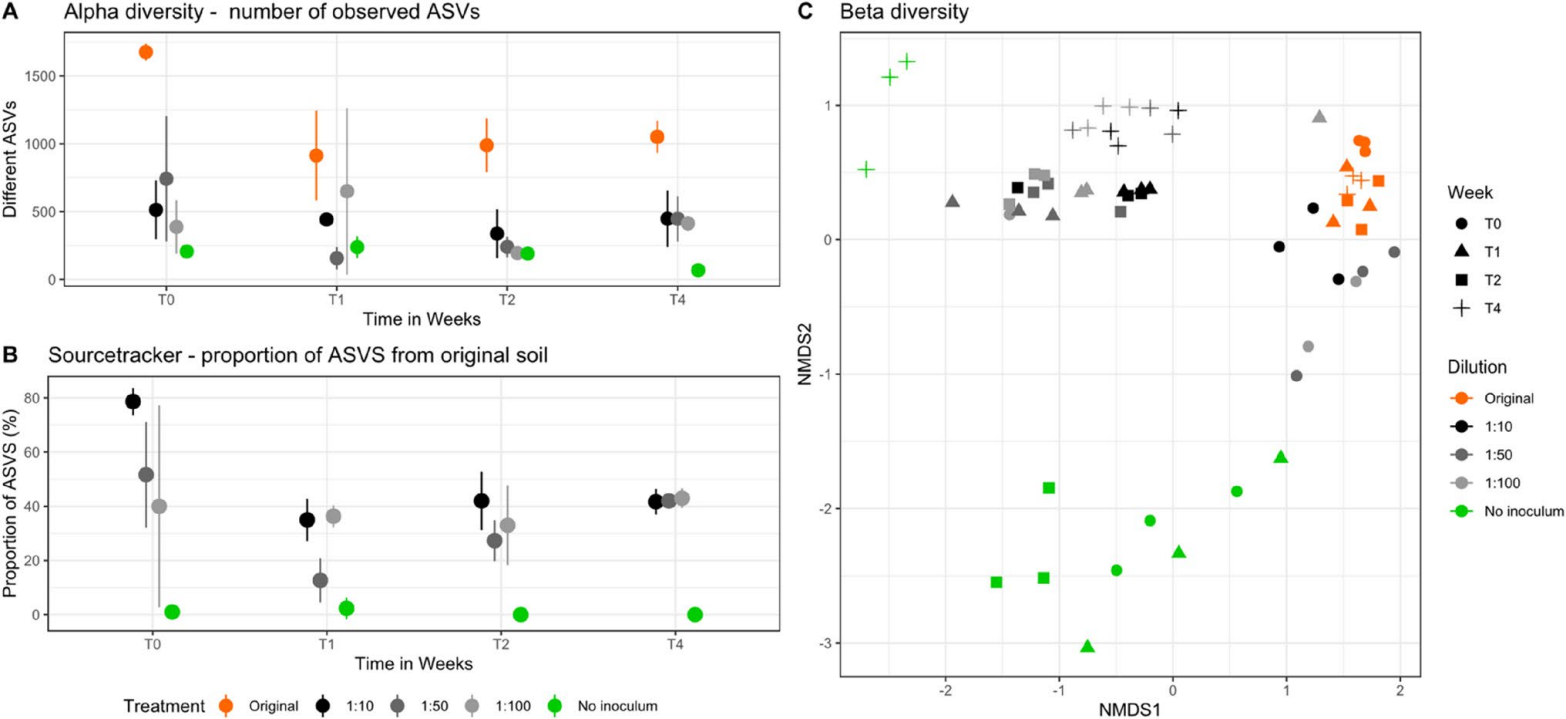
**A** Alpha diversity of prokaryotes shown as the number of observed ASVs at T0, T1, T2 and T4. **B** Sourcetracker analysis showing the percentage of ASVs in the diluted samples coming from the original soil over the experimental period (T0–T4). Shown are mean values of three replicates and error bars represent standard deviations. **C** NMDS plot based on Bray-Curtis dissimilarity matrix based on ASVs. Shown is the original soil (orange), 1:10 dilution (black), 1:50 dilution (dark grey), 1:100 dilution (light grey), and non-inoculated (green) soils

As shown in Fig. 2A, both time and treatment had significant effects on alpha diversity (ANOVA *p* = 2.81*10^9^ for dilution effects and *p* = 5.68*10^5^ for time effects). As observed for the abundance, alpha diversity of prokaryotes expressed as number of observed ASVs was stable in the original soil and ranged between 1676 and to 913. The post hoc test indicated significant differences between T0 and T2 (*p* = 0.0061539) as well as treatment specific differences between the original soil and all other treatments (*p* < 0.00001 – Table S2). Diversity was significantly lower in all inoculation treatments compared to original soil ranging from 240 at T2 to 413 at T4 independent from the biomass of the inoculum. In the non-inoculated treatment, the number of observed ASVs was lowest after 4 weeks (T4) of incubation and reached a value of 68 observed ASVs only, which was accompanied by a strong additional drop in Evenness values (Fig. S4). The diversity patters can by mostly attributed to the bacteria community, which composed 98.8% of the assigned reads, while archaea had 1.2%. The diversity pattern can, however, also be observed in the archaeal community (Fig. S6 and Table S3).

### Recovery of Prokaryotic Community Composition After Reinoculation of Sterilized Soil

**Fig. 3:**
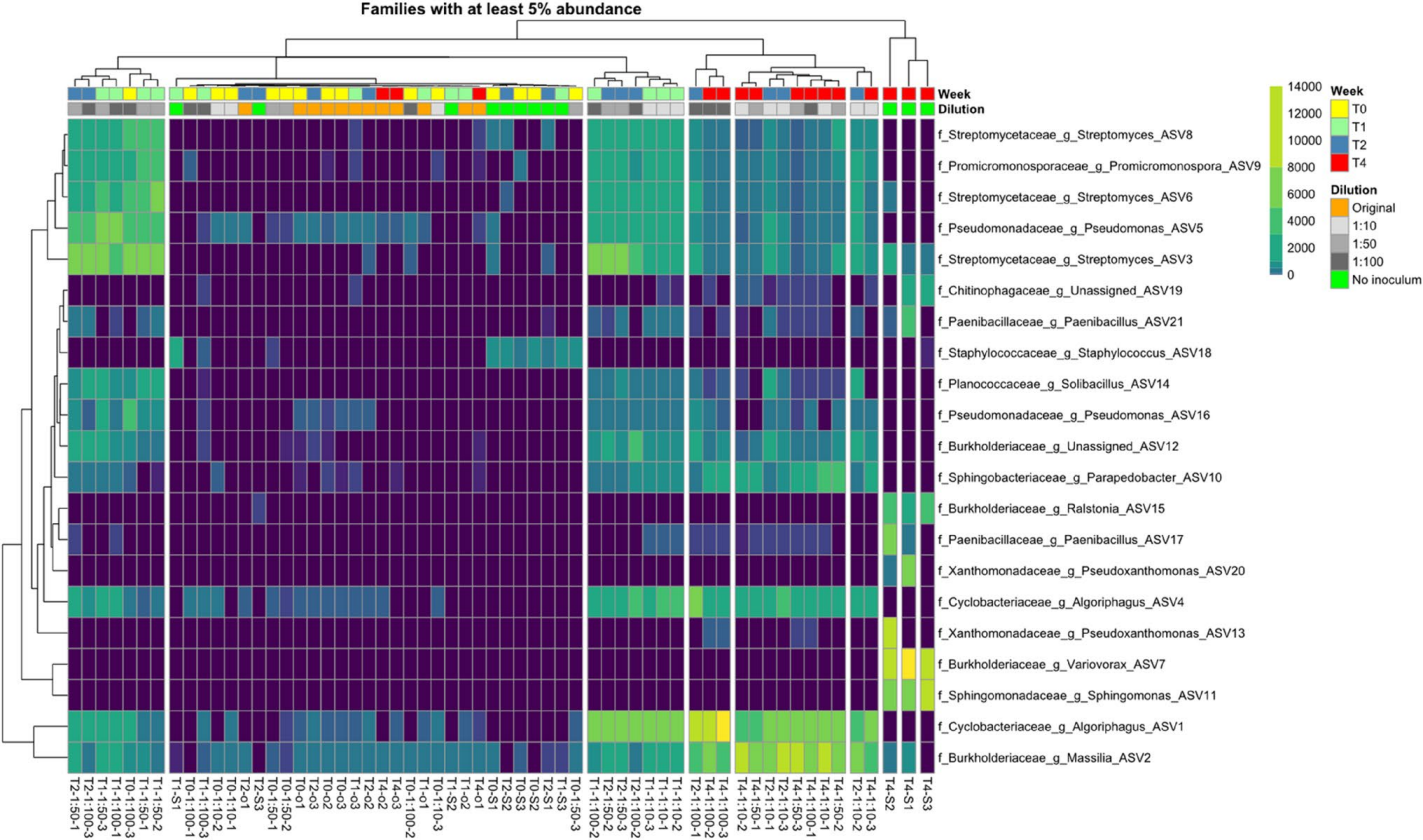
Heatmap and dendrogram showing the relative abundance of dominant ASVs annotated at family and genus level. Depicted ASVs needed to exceed 5% relative abundance in at least one sample. The dendrogram was achieved by applying the Ward’s Hierarchical Clustering. The color codes depict absolute abundance of reads assigned to the given ASVs

The Sourcetracker based mapping of ASVs (Fig. 2B) from the original soil at T0 towards the disturbed and re-inocculated soils demonstrated that ASVs in the inoculated disturbed soils derived from the imoculum. Interestingly, differences in shared ASVs were observed in response to the different inoculation treatments used. For example, at T0, the 1:10 treatment shared 78% of ASVs with the original soil, the 1:50 treatment shared 52%, and the 1:100 treatment shared 40%, while the non-inoculated soil showed no overlap. This result was further confirmed by beta-diversity analysis (Fig. 2C) and hierarchical clustering methods (Fig. 3). The NMDS plot indicated, that at T0, the original soil and the soil inoculation treatments were most similar, except for the non-inoculated soil treatment, which separates from all other T0 samples. The initial load of inoculum impacted the reproducibility of the initial colonization. The 1:10 treatment showed a standard deviation of 5.0% at week 0 based on Sourcetracker analysis, while the 1:50 and 1:100 treatments showed standard deviations of 19.5% and 37.2%, respectively. At T4, all dilution levels converged to a similar share of 42% of ASVs deriving from original soil. The composition of all communities in the inoculated soils significantly differed at T4 from the community composition at T0 (PERMANOVA *p* = 0.006), but no clear pattern in response to the different inoculation densities were observed.

**Fig. 4:**
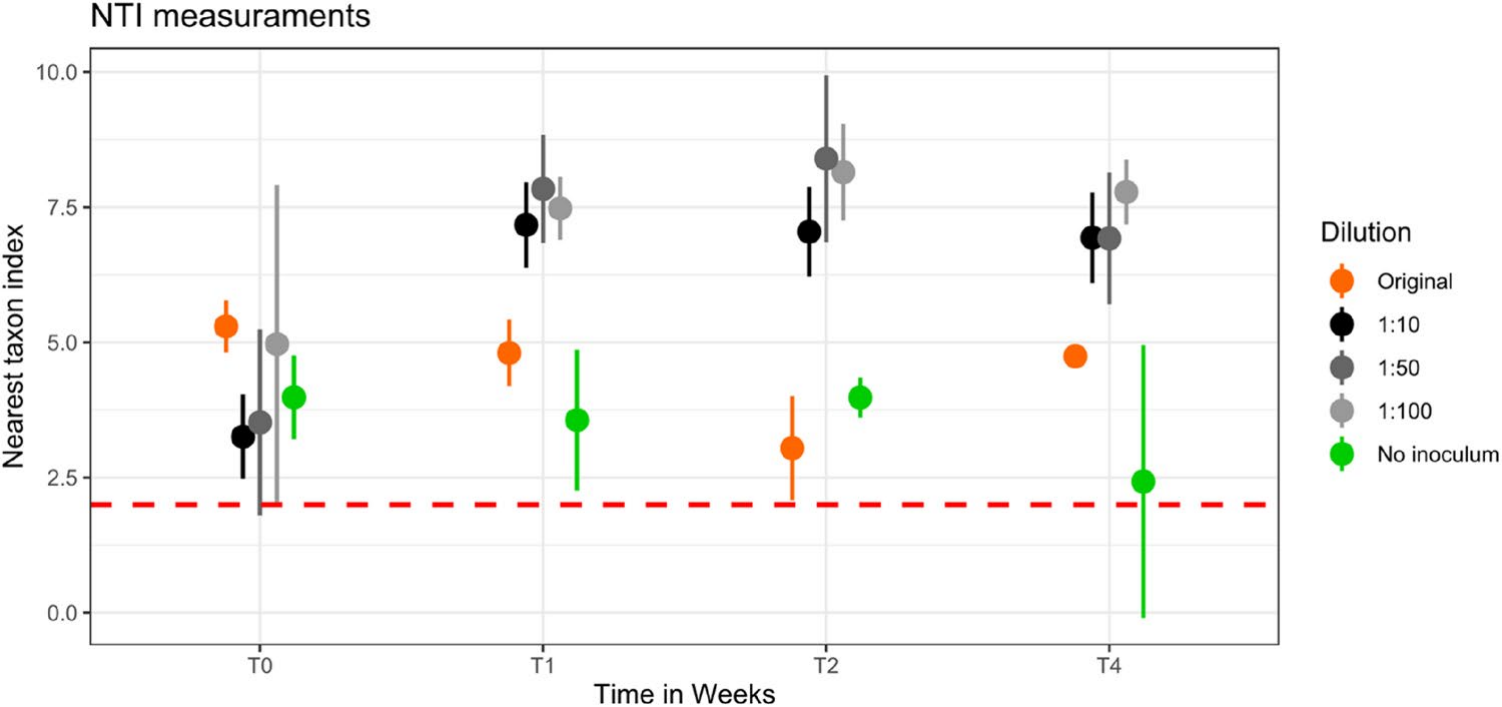
Nearest taxon index measurements built over phylogenetic tree from sequenced samples. The results show that all the samples were clustered above the threshold of 2 (red dashed line), indicating strong phylogenetic signal on all the samples over time. Diluted samples show higher NTI values.

The hierarchical clustering of communities shown in the dendrogram of Fig. 3 further supports that observation. One cluster is represented by samples of the non-inoculated treatment, which is dominated by ASVs assigned to different genera of *Burkholderiaceae* namely *Variovorax* sp. and *Ralstonia* sp., and to specific genera of *Sphingomonadaceae*, *Chitinophagaceae*, *Paenibacillaceae*, and *Xanthomonadaceae*, which are *Sphinogmonas* sp., *Paenibacillus* sp., and *Pseudoxanthomonas* sp. However, this community significantly differs from the other two clusters. Cluster 2 represented samples of the dilution treatments at T1 and the original soils, which showed an even distribution of ASVs. The dilution samples at T1 are dominated by ASVs assigned to *Cyclobacteriaceae* genus *Algoriphagus* sp., *Promicromonosporaceae* genus *Promicromonospora* sp., *Pseudomonadacaea* genus *Pseudomonas*, and *Streptomycetaceae* genus *Streptomyces* sp. The third cluster includes all dilution treatments at T4, but also those from T2 and is characterized by samples, which have a high relative abundance of ASVs assigned to *Burkholderiaceae*, *Cyclobacteriaceae* genus *Algoriphagus* sp. and *Sphingobacteriaceae* genus *Parapedobacter* sp., *Paenibaciliaceae* genus *Paenibacillus* sp. Compared to cluster 1 another genus of *Burkholderiaceae* was identified in cluster 2, which was *Massilia* sp. The dominance of those families indicates an initial bloom of copiotrophic bacteria, which decline during the subsequent incubation period. The results of NTI calculation revealed that changes in community composition are mostly deterministic, as all samples had an NTI value > 2 (Fig. 4), which indicates “niche-based” deterministic mechanisms. Nevertheless, the diluted samples always exceeded the non-inoculated soil samples, indicating a stronger phylogenetic clustering as a result of the inoculation.

## Discussion

### Biomass Recovery Is Driven by Few Taxa

Our data indicated that the recovery of microbial biomass (Fig. 1) was different for bacteria and archaea and was driven by few taxa, which quickly increased in relative abundance (Fig. 3). Although the initial microbial load was consistent to the dilution (highest abundance in original soil, followed by 1:10, 1:50, 1:100, and non-inoculated soil treatments), bacterial abundance recovered quicker than the archaeal abundance, while diversity recovery followed similar pattern. Previous data [32] indicate that colonization is driven for bacteria in nutrient-rich habitats, but for archaea more under oligotrophic conditions [32]. The soil in our study can be considered as nutrient rich, as the process of autoclaving releases dissolved nutrients from dead cells into the soil. The DOC concentrations after autoclaving (Fig. S5) revealed a significant increase, which was also been previously observed [33]. The flush of carbon was most likely derived from dead cells [34]. Microbial necromass was already shown to be an important player for soil nutrient turnover [35]. Furthermore, the Jena Experiment is built on a previous agricultural site, which was frequently fertilized [20]. This in combination with the release of organic carbon as a result of autoclaving might have favored fast-growing bacteria.

In our study, the most dominant bacteria has been classified as copiotroph for example Burkholderiaceae, Sphingomonadaceae, and Xanthomonadaceae [36] able to use organic carbon and nitrogen for quick growth. In contrast to bacteria, archaea recovered slower and never exceeded the abundance of the original soil. Interestingly, this was not reflected by the diversity development of bacteria and archaea, which revealed a similar pattern, indicating that the abundance effects is most likely dedicated to slower growth of the archaeal species.

The analysis of the community composition revealed that prokaryotic growth was not a uniform response over the whole community, but only of some fast-growing taxa mostly dominated by bacteria. We had originally hypothesized that the initial weeks are dominated by spore forming microbes, which are widely spread as spore bank in soil [33]. However, this could be only partly verified. In the non-inoculated soil, *Paenibacillus* sp. was indeed highly dominant but surprisingly only at T4. In the dilution treatments an initial *Streptomyces* bloom was observed at T1. This observation indicates that even though spores might be able to survive extreme conditions as extreme heat, their increase in abundance also depends on interactions with other microbes. In this respect, *Streptomyces* sp. might be especially successful, as they are well known as producers of different antibiotics [34], which displays a selective advantage during colonization of new habitats and occupation of empty niches. Moreover, this family comprises many metabolic versatile taxa [35, 37]. The combination of carbon consumption, antibiotic production, and denitrification is likely responsible for successful colonization of inoculated soils by *Streptomyces* sp. The dominance of Actinobacteria, to which *Streptomyces* belong, was also reported in a study of Delmond et al. (2014) as being highly abundant after soil sterilization and reinoculation and being positively correlated with carbon availability, which was also increased in our study (DOC in Fig. S6). Additionally, the first week of community development in inoculated soils was further dominated by *Cyclobacteriaceae, Pseudomonadaceae*, and *Burkholderiaceae*. *Cyclobacteriaceae* are abundant in soil environments and successful in processing a wide range of polysaccharides due to a rich repertoire of carbohydrate-active enzymes [38]. *Burkholderiaceae* consist of a metabolic diverse range of taxa [39] including denitrifying [40] and plant growth promoting strains.

Denitrification has been often observed in nutrient-rich environments [37], as is the case of soil used in our study, indicating a very early establishment of microbiota driving key processes of N cycling including mineralization of N and nitrification, resulting in the needed nitrate for denitrification. The abundance *Massilia* and *Streptomyces* genera in the inoculated soils indicates that microbial community on these treatments sustained a better metabolization of complex carbon forms in the soil, as chitin [41] and cellulose, speeding up the assimilation of carbon by the community. Additionally, *Pseudomonas* sp. was highly abundant. This genus is present in a variety of environments from soil to plant and animal tissue [42] and contains both plant pathogens and plant growth promoting bacteria. The distinction of plant growth promoting or pathogenic takes often place on strain level [42].

If no inoculum was applied, soil recolonization was much slower, much more random and resulted in an altered community composition. This difference in community composition and recovery rate stresses the positive effect of inoculation to re-establish original community even in small amounts, i.e., 1:100 dilution. Evidence shows that rarefying microbial communities can impact ecosystem functions and only extreme dilutions can lead to the inability from microbial community to establish over time [12].

### Community Composition Converges if Inoculum Is Given

The beta diversity analysis and Sourcetracker results demonstrated that inoculation of sterilized soils resulted in a prokaryotic community similar to the original soil and that the degree of overlap was further modulated by amount of inoculum. Considering the proportion of relic DNA in the original soil, the overlap might be even higher [22]. However, the following recovery of prokaryotic biomass caused a drop in alpha diversity (Fig. 2A). The diversity of original soil was also never exceeded during the incubation by any of the treatments, which underlines that initial microbial bloom in the dilutions was dominated by a few microbial groups, as discussed in previous sections. Diversity seemed to randomly fluctuate over time on non-inoculated soils and on soils which received less inoculate biomass (1:50 and 1:100). Nevertheless, after 4 weeks all microbial communities of the soils receiving an inoculum converged and were not significantly different from each other anymore (PERMANOVA *p* = 0.317). However, original community composition was only partly resembled; indicating that regardless of initial inoculation load, the inoculated soils had the potential to reproducibly recolonize the non-inoculated one, but original diversity was not reached at least during the incubation period. All the inoculated soils stabilized in an intermediary stage in terms of diversity and community composition between original and non-inoculated disturbed soil (Fig. 2A). These findings are strongly supported by the NTI measurements, which clearly points to microbial community’s deterministic adaptation. This was further demonstrated by the convergent behavior of communities regardless of inoculation amount. Interestingly, the NTI values tripled from week 0 to week 1 in the dilutions, while it remained relatively stable not just in the original soil, but also in the non-inoculated soil samples (Fig. 3). This data shows that microbial community present in the inoculum actively reacts to environmental filtering, strengthening the phylogenetic signal. These findings corroborate the theory that phylogenetically closer taxa would prefer similar habitats and perform similar ecological processes [43], here selected by its ability to utilize easily available carbon and other nutrient sources and quickly occupy empty niches.

The effect of soil inoculation on recovering microbial diversity has already been reported in a similar study [11]. In this study, 2 g of soil from different countries was inoculated in 30 g of sterilized soil (1:15 proportion), leading to similar final microbial communties after an incubaction period of 6 months. Those findings corroborate with our results, in a way that soil as inoculum harbor enough microbial diversity to populate new environments according to environmental filtering [1, 44]. In this sense, the community establishment limitation is not the diversity in the initial inoculum, as stated by Deltmont et al. (2014) or the load of the inoculum, which we tested in our experiment, but rather the environmental filter imposed to this community. In terms of microbial diversity recovery, the initial loss of diversity and subsequencial recovery was also oberved in chronosequence studies by Li et al. [8] and Jurburg et al. [45], where microbial diveristy decreased after an initial disturbance, being dominted by a few surviving taxa. Jurburg et al. [45] also reported microbial community development to be ruled by niche processes.

When we studied the natural development of microbial communities in non-inoculated soil, the recovery was much slower, less reproducible, and resulted in an altered community composition. The competition with indigenous microbes is lower in autoclaved treatment, thus empty niches can be randomly colonized. Potential sources for the initial community might be prokaryotes resistant to soil autoclaving procedure as it might be the case for *Paenibacillaceae* or airborne prokaryotes. In previous studies, Firmicutes, Proteobacteria, and Bacteriodetes were frequently detected in different bioaerosols [46, 47]. However, their settlement might be random, in low abundance and thus causing high fluctuation in community dynamics.

Interestingly, the Sourcetracker mapping of shared ASVs indicated that initial microbial load followed the dilution pattern, being 1:10 dilution, the one with higher percentage of shared ASVs (79%), followed by 1:50 (52%), and 1:100 (40%) (Fig. 2B). Considering that even for 1:100 dilution, 0.15 g of original soil was added, which was similar in bacterial and archaeal diversity compared to original soil and the other two dilutions, this result is surprising, but indicates that the amount of applied microbiota mainly for the less abundant ones is an important factor for recolonization. The numbers of shared ASVs might be even higher, considering the amount of relic DNA present in the original soil [22], which in our case was approximately 23%.

## Conclusion

Despite the study of deterministic and stochastic colonization being a well-known field in ecology and fairly well covered in microbial ecology [6, 48, 49], proofs of principle and case studies are still poorly explored. Currently, literature indicate that environmental filtering pressure might vary when physicochemical parameters change [50] and deterministic community development seems to be predominant over stochastic ones [17, 48, 51]. Together with the data from Delmont et al. (2014), our study demonstrated that soil physicochemical conditions overshadow initial inoculum load and composition as determining factor for community establishment. Such knowledge has to be taken in consideration during the design of inoculation experiments [52, 53] and indicated that soil management might be a clever approach to enrich certain microbial taxa [54], [55].

In summary, our data indicates that microbial community assembly in sterilized and re-inocculated soils occurs in deterministic ways, being ruled by “niche-based” interactions. In agreement with our expectations, microbial inoculation drives soil colonization, however unable to fully recover initial microbial composition and diversity, leading in the end to the dominance of different microbial taxa. Colonization was clearly driven by bacteria compared to a stable archaeal community. The changes in environmental filtering can be mostly attributed to physicochemical changes in the soil after autoclaving. Although still being ruled by niche processes, the non-inoculated soil did not have a clear colonization pattern, reinforcing the importance of inoculation to stochastic colonization. Overall, our findings may help to better understand the process of microbial establishment in soil communities, as well as the limitations of soil microbiome manipulation, which can have important practical implications during soil restoration.

### Supplementary Information

ESM 1 (DOCX 1467 kb)

## Data Availability

The sequencing datasets generated during the current study are available in the NCBI sequencing read archive under the bioproject number PRJNA937438.
