# Supplementary material for: Deterministic Development of Soil Microbial Communities in Disturbed Soils Depends on Microbial Biomass of the Bioinoculum
Source: Microb Ecol. 2023 Aug 25;86(4):2882–93. doi: 10.1007/s00248-023-02285-9 (PMC10640511; doi:10.1007/s00248-023-02285-9)
Supplement: Supplementary file 1 — (DOCX 1467 kb) [file 248_2023_2285_MOESM1_ESM.docx]

**Supplementary Material:**


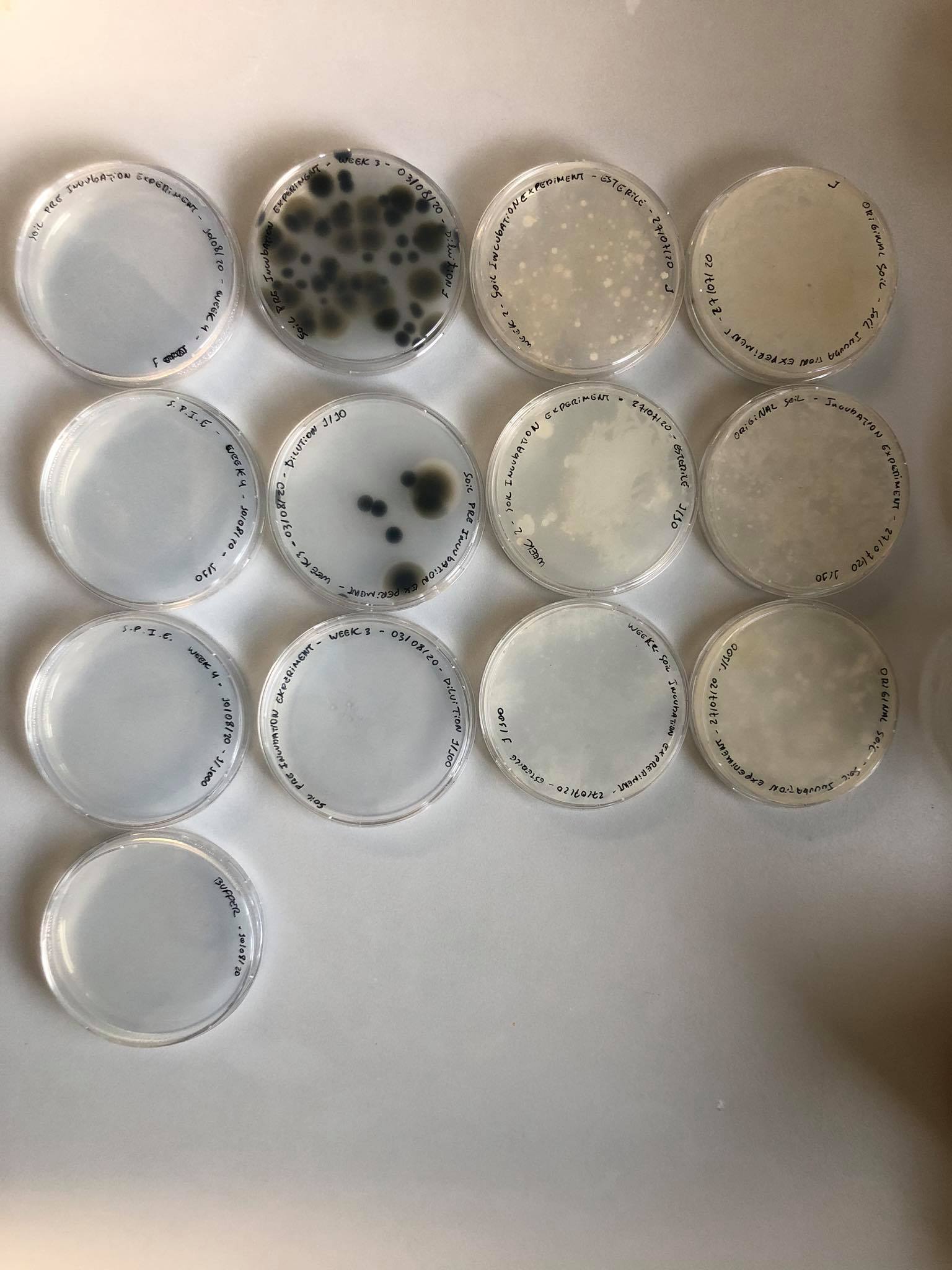


1st week

2nd week

3rd week

4th week and buffer control

Fig. S1: Microbial growth during the soil autoclavation over the weeks. 1g of autoclaved soil was resuspended in 0.8% NaCl2 solution. 100 µl of resuspended soil was plated in R2A agar plates also in dilutions 1:10 and 1:100. In the picture we show the last dilution to have growth in each of the time points. At week 4, no microbial growth could be observed.

*Table S1: Table displaying the ANOVA and Post Hoc test (Tukey test) results for qPCR copy number for both bacterial and archaeal 16S rRNA gene. Fitting order for ANOVA was CopyNumber ~ Dilution + Time, using aov function in base R. Significant effects are labelled p < 0.05, p < 0.005 or p < 0.0005 are labelled with *, ** or ***.*

| **Anova** | | | | | | | | | | | |
| --- | --- | --- | --- | --- | --- | --- | --- | --- | --- | --- | --- |
| **Bacteria** | | | | | | **Archaea** | | | | | |
|  | Df | Sum Sq | Mean Sq | F value | Pr(>F) |  | Df | Sum Sq | Mean Sq | F value | Pr(>F) |
| Dilution | 4 | 5,58E+21 | 1,39E+21 | 3.918 | 0.007** | Dilution | 4 | 8,95E+15 | 2,24E+15 | 40.927 | 1.79e-15*** |
| Time | 3 | 5,60E+21 | 1,87E+21 | 5.247 | 0.003** | Time | 3 | 2,74E+14 | 9,15E+13 | 1.673 | 0.184 |
| Residuals | 52 | 1,85E+22 | 3,56E+20 |  |  | Residuals | 52 | 2,84E+15 | 5,47E+13 |  |  |
| **Tukey Post Hoc Test** | | | | | | | | | | | |
| **Bacteria** | | | | | | **Archaea** | | | | |  |
| **Dilution** | | | | |  | **Dilution** | | | | |  |
|  | diff | lwr | upr | p adj |  |  | diff | lwr | upr | p adj |  |
| 1:100-1:10 | -3,9E+08 | -1,1E+09 | 3,03E+08 | 0.516 |  | 1:100-1:10 | -227324.54 | -497097.5 | 42448.42 | 0.137 |  |
| 1:50-1:10 | -1,3E+08 | -8,2E+08 | 5,57E+08 | 0.983 |  | 1:50-1:10 | -162008.58 | -431781.5 | 107764.39 | 0.444 |  |
| No inoculum-1:10 | -7,9E+08 | -1,5E+09 | -1,1E+08 | 0.016* |  | No inoculum-1:10 | -330206.29 | -599979.3 | -60433.33 | 0.009* |  |
| Original-1:10 | -6,8E+08 | -1,4E+09 | 10503657 | 0.056 |  | Original-1:10 | 747822.45 | 478049.5 | 1017595.42 | 0.000*** |  |
| 1:50-1:100 | 2,53E+08 | -4,3E+08 | 9,42E+08 | 0.835 |  | 1:50-1:100 | 65315.96 | -204457.0 | 335088.93 | 0.959 |  |
| No inoculum-1:100 | -4,1E+08 | -1,1E+09 | 2,79E+08 | 0.455 |  | No inoculum-1:100 | -102881.75 | -372654.7 | 166891.21 | 0.817 |  |
| Original-1:100 | -2,9E+08 | -9,8E+08 | 3,96E+08 | 0.750 |  | Original-1:100 | 975147.00 | 705374.0 | 1244919.96 | 0.000*** |  |
| No inoculum-1:50 | -6,6E+08 | -1,4E+09 | 25599410 | 0.064 |  | No inoculum-1:50 | -168197.71 | -437970.7 | 101575.25 | 0.406 |  |
| Original-1:50 | -5,5E+08 | -1,2E+09 | 1,42E+08 | 0.181 |  | Original-1:50 | 909831.03 | 640058.1 | 1179604.00 | 0.000*** |  |
| Original-No inoculum | 1,17E+08 | -5,7E+08 | 8,05E+08 | 0.989 |  | Original-No inoculum | 1078028.75 | 808255.8 | 1347801.71 | 0.000*** |  |
| **Time** | | | | |  | **Time** | | | | |  |
|  | diff | lwr | upr | p adj |  |  | diff | lwr | upr | p adj |  |
| T1-T0 | 7,24E+08 | 1,46E+08 | 1,3E+09 | 0.009* |  | T1-T0 | 93064.98 | -133566.58 | 319696.54 | 0.697 |  |
| T2-T0 | 1,94E+08 | -3,8E+08 | 7,73E+08 | 0.809 |  | T2-T0 | -57261.20 | -283892.76 | 169370.36 | 0.908 |  |
| T4-T0 | 6,58E+08 | 79487303 | 1,24E+09 | 0.019* |  | T4-T0 | 107013.97 | -119617.59 | 333645.54 | 0.596 |  |
| T2-T1 | -5,3E+08 | -1,1E+09 | 48517645 | 0.084 |  | T2-T1 | -150326.18 | -376957.74 | 76305.38 | 0.304 |  |
| T4-T1 | -6,6E+07 | -6,4E+08 | 5,12E+08 | 0.990 |  | T4-T1 | 13948.99 | -212682.57 | 240580.55 | 0.998 |  |
| T4-T2 | 4,63E+08 | -1,1E+08 | 1,04E+09 | 0.158 |  | T4-T2 | 164275.17 | -62356.39 | 390906.73 | 0.231 |  |

**
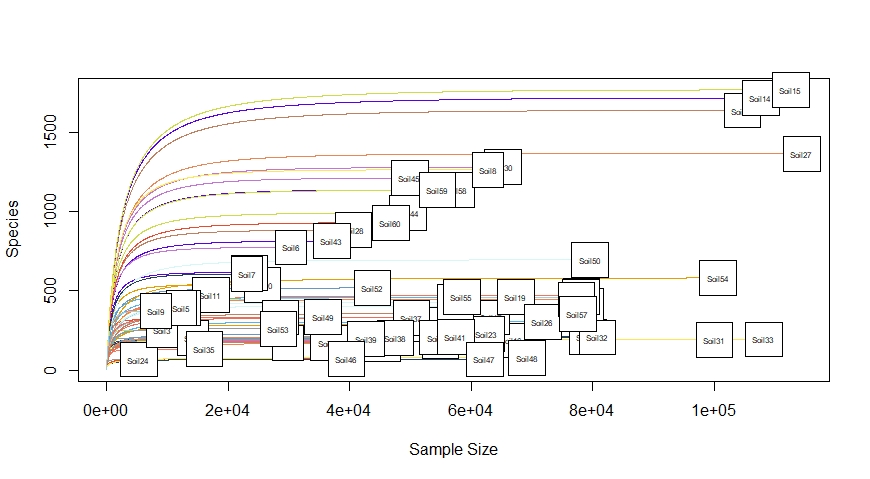
**

Fig. S2: Rarefaction curve built over the ASV table using rarecurve command from Vegan (version 2.6.2) package on R (step=50, cex=0.5).

**
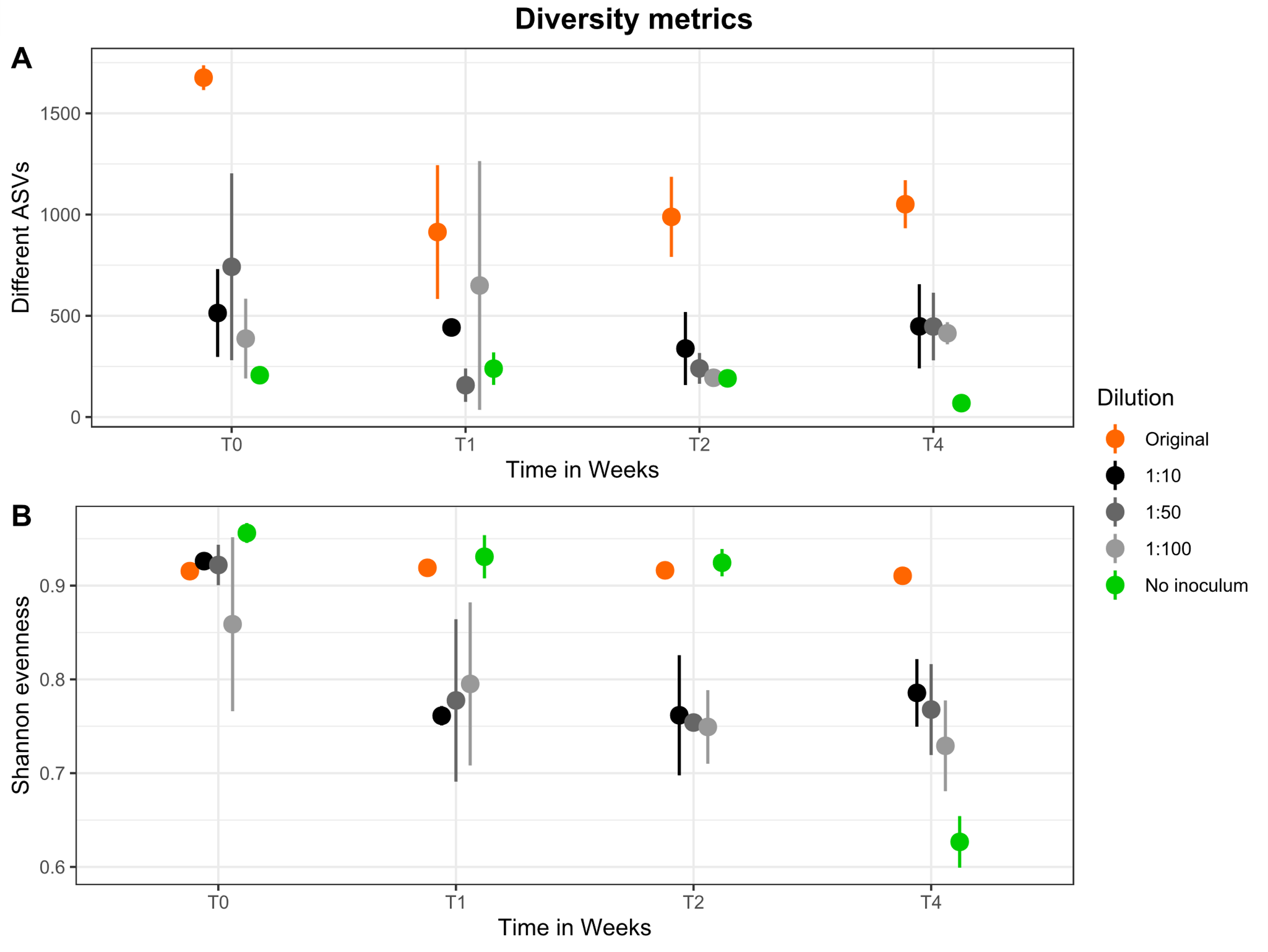
**

Fig. S3: Number of observed ASVS (A) and Shannon evenness (B) measurements calculated over the ASVs table from metabarcoding sequencing for prokaryotes.

*Table S2: Table displaying the ANOVA and Post Hoc test (Tukey test) results for alpha diversity (calculated based on the number of observed ASVs). Fitting order for PERMANOVA was CopyNumber ~ Dilution + Time, using aov function in base R. Significant effects are labelled p < 0.05, p < 0.005 or p < 0.0005 are labelled with *, ** or ***.*

| **Anova** | | | | | |
| --- | --- | --- | --- | --- | --- |
|  | Df | Sum Sq | Mean Sq | F value | Pr(>F) |
| Dilution | 4 | 31.98 | 7.996 | 17.848 | 2.81e-09 *** |
| Time | 3 | 12.33 | 4.109 | 9.171 | 5.68e-05 *** |
| Residuals | 52 | 23.30 | 0.448 |  |  |
|  |  |  |  |  |  |
| **Tukey Post Hoc Test** | | | | | |
| **Dilution** | | | | |  |
|  | diff | lwr | upr | p adj |  |
| 1:100-1:10 | -2.441.667 | -3.133.736 | 26.454.031 | 0.999 |  |
| 1:50-1:10 | -3.891.667 | -3.278.736 | 25.004.031 | 0.995 |  |
| Original-1:10 | 72.175.000 | 4.327.930 | 101.070.697 | 0.000*** |  |
| Sterile-1:10 | -25.925.000 | -5.482.070 | 2.970.697 | 0.098 |  |
| 1:50-1:100 | -1.450.000 | -3.034.570 | 27.445.697 | 0.999 |  |
| Original-1:100 | 74.616.667 | 4.572.097 | 103.512.364 | 0.000*** |  |
| Sterile-1:100 | -23.483.333 | -5.237.903 | 5.412.364 | 0.162 |  |
| Original-1:50 | 76.066.667 | 4.717.097 | 104.962.364 | 0.000*** |  |
| Sterile-1:50 | -22.033.333 | -5.092.903 | 6.862.364 | 0.213 |  |
| Sterile-Original | -98.100.000 | -12.699.570 | -69.204.303 | 0.000*** |  |
| **Time** |  |  |  |  |  |
| diff | lwr | upr | p | p adj |  |
| T1-T0 | -224.733.333 | -4.674.810 | 1.801.438 | 0.079 |  |
| T2-T0 | -314.666.667 | -5.574.144 | -7.191.895 | 0.006* |  |
| T4-T0 | -219.466.667 | -4.622.144 | 2.328.105 | 0.089 |  |
| T2-T1 | -89.933.333 | -3.326.810 | 15.281.438 | 0.759 |  |
| T4-T1 | 5.266.667 | -2.374.810 | 24.801.438 | 0.999 |  |
| T4-T2 | 95.200.000 | -1.475.477 | 33.794.771 | 0.726 |  |


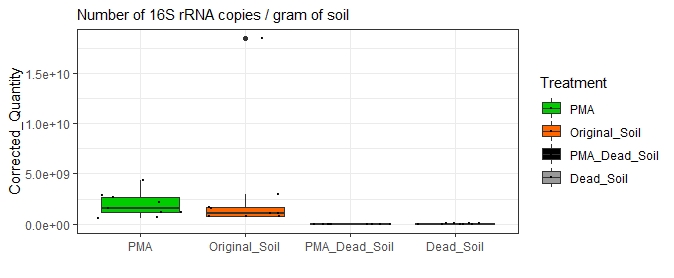


Fig. S4: PMA PCR performed with Original soil used on the inoculation. Boxes show both minimum and maximum values in both edges, 25th Percentile, Median and 75th percentile. PMA treatment had average number 16S rRNA copies of 1.905.079.047 (median value of 1.553.144.704 copies), while Original, untreated soil had 3.224.934.322 (median value of 1.088.152.000 copies). The “dead” soil control was meant to access the limitation of PMA to fully remove DNA from samples and how it impacts on the qPCR amplification. While the “dead” soil without PMA treatment had 21.401.234 reads in average, PMA treated “dead” soil had 7.254.347, indicating that 37% of the DNA remains in the soil. Considering an efficiency of 63%, and that the PMA treatment removed 38% of the DNA on the Original soil, we can estimate that 23,9% of the DNA present in the Original soil derivates from dead or damaged cells


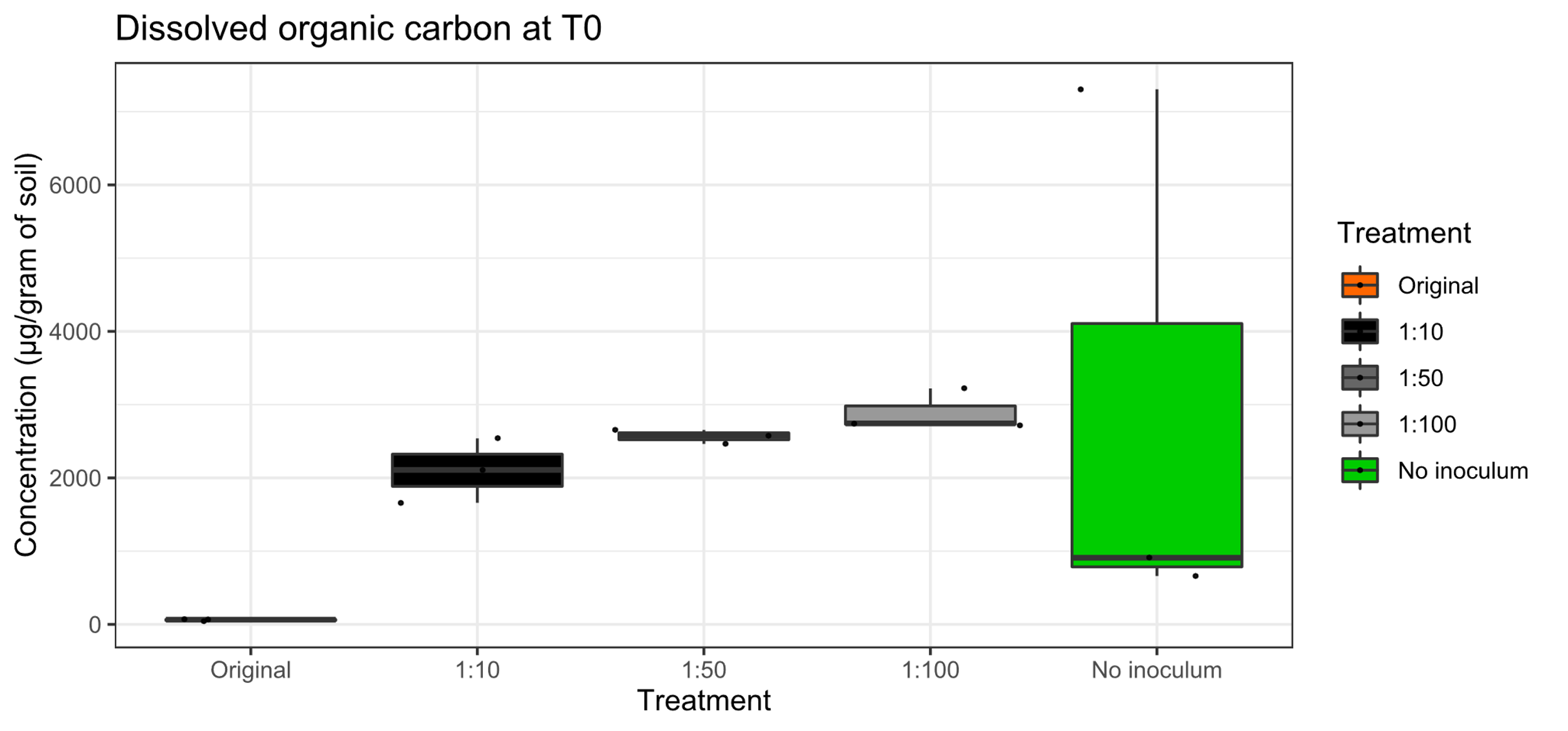


*Fig. S5: Dissolved organic carbon at time point week 0.*


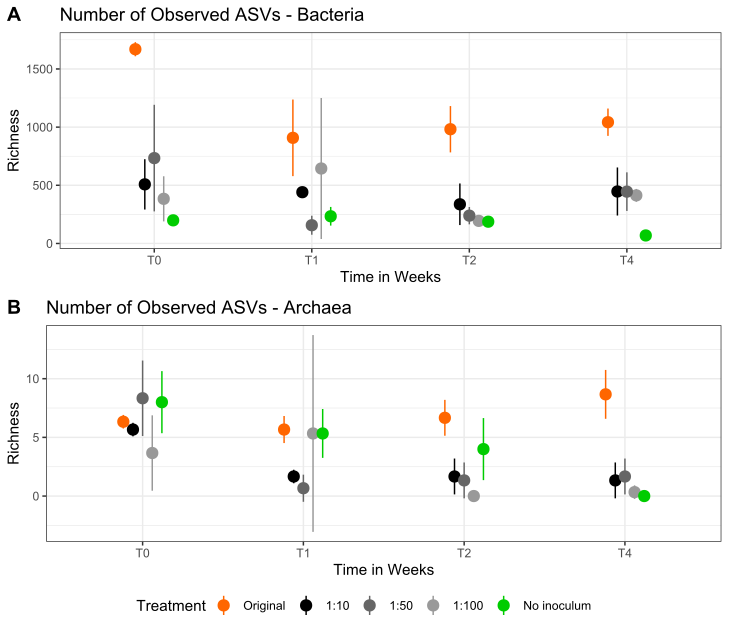


Fig. S6: Diversity (Number of Observed ASVs) of Bacterial and Archeal reads along the experiment´s duration. The primeir pair used in this study (The Earth Microbiom project´s Eukariotic primer) target both Archaea and Bacteria, so the bacterial and archaeal assinged reads were filtered out of the total dataset using phyloseq. From the 7820 ASVs in the dataset (distributed among 3.224.377 reads), 56 ASVs (38.998 reads) were assigned to Archaea, totalizing 1.2% of the annotated reads. We used the number of observed ASVs as alpha diversity metric. Overall diversity follows the patters of the combined community, with significant effects of Dilution and Time (see supplementary table S3 for statistical significances).

| **Anova** | | | | | | | | | | | | |
| --- | --- | --- | --- | --- | --- | --- | --- | --- | --- | --- | --- | --- |
| Bacteria | | | | | | Archaea | | | | | | |
|  | Df | Sum Sq | Mean Sq | F value | Pr(>F) |  | Df | Sum Sq | Mean Sq | F value | Pr(>F) |  |
| Dilution | 4 | 6650023 | 1662506 | 26.835 | 4.2e-12*** | Dilution | 4 | 165.1 | 41.27 | 4.877 | 0.00205** |  |
| Week | 3 | 785774 | 261925 | 4.228 | 0.0095** | Week | 3 | 147.9 | 49.31 | 5.827 | 0.00165** |  |
| Residuals | 52 | 3221549 | 61953 |  |  | Residuals | 52 | 440.0 | 8.46 |  |  |  |
| **Tukey Post Hoc Test** | | | | | | | | | | | | |
| Bacteria | | | | | | Archaea | | | | | | |
| Dilution | diff | lwr | upr | p adj |  | Dilution | diff | lwr | upr | p adj |  |  |
| 1:100-1:10 | -2.416.667 | -3.113.077 | 26.297.432 | 0.9992683 |  | 1:100-1:10 | -0.2500000 | -36.057.466 | 3.105.747 | 0.9995481 |  |  |
| 1:50-1:10 | -3.933.333 | -3.264.743 | 24.780.766 | 0.9950984 |  | 1:50-1:10 | 0.4166667 | -29.390.799 | 3.772.413 | 0.9966470 |  |  |
| No inoculum-1:10 | -26.100.000 | -5.481.410 | 2.614.099 | 0.0913721 |  | No inoculum-1:10 | 17.500.000 | -16.057.466 | 5.105.747 | 0.5838653 |  |  |
| Original-1:10 | 71.750.000 | 4.303.590 | 100.464.099 | 0.0000000 |  | Original-1:10 | 42.500.000 | 0.8942534 | 7.605.747 | 0.0065186 |  |  |
| 1:50-1:100 | -1.516.667 | -3.023.077 | 27.197.432 | 0.9998845 |  | 1:50-1:100 | 0.6666667 | -26.890.799 | 4.022.413 | 0.9800060 |  |  |
| No inoculum-1:100 | -23.683.333 | -5.239.743 | 5.030.766 | 0.1516780 |  | No inoculum-1:100 | 20.000.000 | -13.557.466 | 5.355.747 | 0.4524360 |  |  |
| Original-1:100 | 74.166.667 | 4.545.257 | 102.880.766 | 0.0000000 |  | Original-1:100 | 45.000.000 | 11.442.534 | 7.855.747 | 0.0034737 |  |  |
| No inoculum-1:50 | -22.166.667 | -5.088.077 | 6.547.432 | 0.2029099 |  | No inoculum-1:50 | 13.333.333 | -20.224.133 | 4.689.080 | 0.7938224 |  |  |
| Original-1:50 | 75.683.333 | 4.696.923 | 104.397.432 | 0.0000000 |  | Original-1:50 | 38.333.333 | 0.4775867 | 7.189.080 | 0.0176278 |  |  |
| Original-No inoculum | 97.850.000 | 6.913.590 | 126.564.099 | 0.0000000 |  | Original-No inoculum | 25.000.000 | -0.8557466 | 5.855.747 | 0.2334345 |  |  |
|  |  |  |  |  |  |  |  |  |  |  |  |  |
| Time |  |  |  |  |  | Time | diff | lwr | upr | p adj |  |  |
| T1-T0 | -22.206.667 | -4.632.888 | 1.915.547 | 0.0814537 |  | T1-T0 | -26.666.667 | -5.485.771 | 0.1524375 | 0.0699752 |  |  |
| T2-T0 | -31.100.000 | -5.522.221 | -6.977.786 | 0.0064944 |  | T2-T0 | -36.666.667 | -6.485.771 | -0.8475625 | 0.0059496 |  |  |
| T4-T0 | -21.546.667 | -4.566.888 | 2.575.547 | 0.0955736 |  | T4-T0 | -40.000.000 | -6.819.104 | -11.808.958 | 0.0023291 |  |  |
| T2-T1 | -8.893.333 | -3.301.555 | 15.228.880 | 0.7622247 |  | T2-T1 | -10.000.000 | -3.819.104 | 18.191.042 | 0.7827191 |  |  |
| T4-T1 | 660.000 | -2.346.221 | 24.782.214 | 0.9998608 |  | T4-T1 | -13.333.333 | -4.152.438 | 14.857.708 | 0.5950701 |  |  |
| T4-T2 | 9.553.333 | -1.456.888 | 33.675.547 | 0.7203382 |  | T4-T2 | -0.3333333 | -3.152.438 | 24.857.708 | 0.9891691 |  |  |

*Table S3: Table displaying the ANOVA and Post Hoc test (Tukey test) results for alpha diversity (calculated based on the number of observed ASVs) for bacterial and archaeal community individually. Fitting order for PERMANOVA was CopyNumber ~ Dilution + Time, using aov function in base R. Significant effects are labelled p < 0.05, p < 0.005 or p < 0.0005 are labelled with *, ** or ***.*
